# Training in Cultural Competence for Mental Health Care: A Mixed-Methods Study of Students, Faculty, and Practitioners from India and USA

**DOI:** 10.1007/s11013-024-09867-3

**Published:** 2024-07-02

**Authors:** Vaishali V. Raval, Baiju Gopal, Pankhuri Aggarwal, Miriam Priti Mohan, P. Padmakumari, Elizabeth Thomas, Aaron M. Luebbe, M. Cameron Hay

**Affiliations:** 1 Miami University, Oxford, OH USA; 2 CHRIST (Deemed to be) University, Bengaluru, India; 3 Department of Psychology, Miami University, 90 N Patterson Ave., Oxford, OH 45056 USA

**Keywords:** Cultural competence, Graduate mental health training, Diversity, India, USA

## Abstract

Although the need to train clinicians to provide effective mental health care to individuals from diverse backgrounds has been recognized worldwide, a bulk of what we know about training in cultural competence (CC) is based on research conducted in the United States. Research on CC in mental health training from different world populations is needed due to the context-dependent nature of CC. Focusing on India and USA, two diverse countries that provide complementary contexts to examine CC, we explored graduate students’, practicing clinicians’, and faculty members’ perspectives regarding CC training they received/provided and future training needs using mixed-methods. The data were collected using focus groups (*n* = 25 groups total: 15 in India, 11 in USA), and a survey (*n* = 800: 450 in India, 350 in USA). Our data highlight the salient social identities in these countries, and the corresponding constituents of CC training. Participants in India described a practical emphasis to their CC training (e.g., learning about CC through life experiences and clinical practice experiences) more so than through coursework, whereas participants in USA described varying levels of coursework related to CC along with practice. Participants in both countries considered enormity of CC as a challenge, while those in the US also identified CC training limited to a white, straight, male perspective, hesitancy in engaging with diversity topics, and limited time and competence of the faculty. Strengths of CC training in India and USA are mutually informative in generating recommendations for enhancing the training in both countries.

## Introduction

In diverse societies, mental health care needs to acknowledge the importance of diverse worldviews, beliefs, and norms, and incorporate this understanding into diagnosis, assessment, and interventions to meet the unique cultural needs of different individuals. When mental health care is provided in a culturally competent manner, health outcomes for individuals with diverse sociocultural identities improve (Bhui et al., 2007). Given the critical significance of cultural competence (CC), programs that train mental health professionals need to incorporate training in CC. Existing research has examined trainee perceptions of CC training (Australia: Geerlings et al., 2014; USA: Green et al., 2009; Gregus et al., 2020), the extent to which CC is practiced (Hansen et al., 2006; Sehgal et al., 2011), and effectiveness of multicultural education on trainees’ CC in USA (systematic review by Benuto et al., 2018; meta-analysis by Smith & Trimble, 2016). Although culturally competent mental health care is relevant for diverse communities worldwide, a bulk of the research on CC in mental health is based primarily on samples from the United States or other countries in the Global North (i.e., Australia, Europe, North America) (Benuto et al., 2018). No published studies have examined whether and how CC is taught in mental health training programs in the Global South (i.e., Africa, Asia, Latin America). Research on CC in mental health training from different world populations is needed because culturally competent practice in part depends on the knowledge of sociocultural identities most relevant in the local communities and contexts where mental health care is provided. Sociodemographic differences based on age, gender, education, and income may have unique meanings in different societies given their distinct social, cultural, political, historic, and economic contexts (Kirmayer, 2012a). Further, exploring CC training in diverse countries can give us insights into how training in CC may be conceptualized and implemented, highlighting the cultural salience of CC training itself with implications for strengthening such training around the world. Focusing on two highly diverse countries, one from the Global North and another from the Global South (USA and India, respectively) that provide differing social, cultural, religious, economic, educational, and healthcare contexts, the current study aimed to describe CC training. Using mixed-methods, we explored the perspectives of three key informants (graduate students, faculty, and practitioners) regarding how they describe learning or teaching CC in graduate psychology training, perceptions of challenges with CC training, and future training needs.

### Various Conceptions and Critiques of Cultural Competence

The concept of CC emerged in the USA in 1980s with the recognition that healthcare systems were poorly designed to meet the needs of diverse patient populations (Betancourt et al., 2003; Kirmayer, 2012a). The needs of individuals from marginalized groups were not appropriately met by healthcare providers with research showing that racial and ethnic minority groups in USA higher unmet mental health needs and lower use of mental health services (Flores, 2010). If clients perceive and experience mental health care providers as unable to understand their values, beliefs, experiences, and needs, and as lacking sensitivity and skills to address their unique cultural experiences, clients may not seek care, and if they do, they may not benefit from the care provided. Training in CC can contribute to improving the quality of mental health care for diverse clients and may reduce health disparities (Betancourt et al., 2003).

From a systemic perspective, within counseling psychology, culturally competent care has been defined as a system that “acknowledges and incorporates—at all levels—the importance of culture, assessment of cross-cultural relations, vigilance toward the dynamics that result from cultural differences, expansion of cultural knowledge, and adaptation of services to meet culturally unique needs” (p. 294, Betancourt et al., 2003). At an individual level, Sue et al.’s (1982) tripartite model of multicultural competence highlights three critical components: awareness, knowledge, and skills. A culturally competent mental health professional demonstrates (a) deep awareness of the influence of one’s own cultural identities on one’s personal values and assumptions, (b) knowledge of family structures, gender roles, values and beliefs, as well as sociopolitical influences on different groups, and (c) skills in delivering culturally competent care (Hansen et al., 2006). Such a professional is also able to shift between their own cultural perspective and those of their clients’ (Lakes et al.,^2016^).

Anthropological critiques highlight that models of CC construe culture as static and homogeneous (Carpenter-Song et al., 2007; DelVecchio Good & Hannah, 2015; Kirmayer, 2012a), which misses diversity of lived experiences within a group created through ongoing interactions between individuals and their multiple communities. Further, because the concept of CC emerged in the USA, ethnic and racial differences that are most salient dimension of diversity in the US have become primary in conceptualizing culture and CC (Kirmayer, 2012a). An additional critique highlights that due to its focus on developing a clinician’s skills, CC does not address structural barriers that individuals experiencing mental health challenges face (e.g., discrimination based on income, race, gender, religion, sexual orientation, and other identities), and thus, a shift from CC to structural competence is recommended that allows systemic and policy-level interventions to improve mental health outcomes (Hansen et al., 2018). Other critiques suggest that competence implies mastery of a domain or skillset, whereas conceptualizing culturally sensitive care as a dynamic and ongoing process is beneficial (Crigger et al., 2006; Isaacson, 2014). Thus, some scholars consider cultural humility as opposed to competence as key in training healthcare professionals (Isaacson, 2014; Kirmayer, 2012b), with the recognition that it is impossible to gain adequate knowledge of all sociocultural identities and intersections of those identities, instead suggesting that it is important to be open to learning, actively listening to others while being aware of one’s own thoughts and reactions. Cultural humility involves being other-oriented as opposed to self-oriented, demonstrating respect, and being able to move away from the tendency to assume one’s own beliefs, values, and worldviews as superior (Hook et al., 2013; Kirmayer, 2012b). Some researchers have termed this a process multicultural orientation as opposed to competence (Owen et al., 2011), and others have described cultural safety, drawing attention to historical and political contexts that lead to health inequities and the responsibility of the health provider to create safe spaces (Curtis et al., 2019; Kirmayer, 2012b). Considering these various perspectives together, we conceptualize CC as a dynamic, socially collaborative life-long process that involves openness and respect for human differences while seeking out knowledge and adapting and acquiring skills in attunement with client’s own understanding and interpretations of themselves and others. We use the term CC because it resonates with health professionals worldwide and across disciplines compared to the other terms that are more recent and less widely used.

### Cultural Competence in Mental Health Training Programs in USA

Individual and cultural diversity is one of the key profession-wide competencies outlined in the Standards for Accreditation for Health Service Psychology programs (graduate programs in clinical, counseling, and school psychology) by the American Psychological Association (APA) (Commision on Accreditation-APA, 2018), and training programs have incorporated mandatory training in CC over the last two decades. Some research has focused on identifying the key elements of CC. In a study involving focus groups at a psychiatric facility, administrators, clinicians, and patients defined CC through both group-based characteristics (i.e., shared beliefs, heritage) and person-centered approaches (e.g., considering individual characteristics and trying to relate to the person). They identified specific strategies clinicians use (i.e., sharing similarities, respecting patient wishes, explaining options) to facilitate CC as well as challenges such as clinician bias, constraints based on time and technology, and patients’ views (Aggarwal et al., 2016a, 2016b). Within health service psychology, Hansen et al. (2000) identified 12 specific practice competencies that fall within the areas of awareness (e.g., of one’s own cultural heritage on values, assumptions, biases), knowledge (e.g., of history of oppression, sociopolitical influences, family structures and gender roles, views about illness and health and help-seeking behavior, culturally salient diagnoses, and assessment procedures) and skills (e.g., to establish rapport, modify assessment tools, design and implement interventions for diverse clients). While these competencies may be included in training programs, based on a survey of professional psychologists, Hansen et al. (2006) found that 86% of the 52 multicultural competencies were not practiced. The researchers concluded that although educators have succeeded in highlighting the importance of CC, training programs need to work harder to implement CC skills.

Based on an early review of available models of CC in mental health care in medical and nursing programs, Bhui et al. (2007) concluded that the curricula focus on a sequential progression from cultural awareness to knowledge to skills. In a more recent review, the content of CC training in health service psychology was found to be similar, focusing on awareness, knowledge, and skills (Benuto et al., 2018). Training methods were also similar including lectures, discussion, experiential, and self-reflective activities (e.g., discussion of clinical cases, role-play, journaling) (Benuto et al., 2018; Bhui et al., 2007) with additional approaches such as cultural immersion activities and contact with individuals with diverse identities mentioned in Benuto and colleagues’ recent review. Methods such as role-play and video demonstration were also considered most helpful by a variety of mental health professionals from different countries in developing CC (Aggarwal et al., 2016a, 2016b).

Some research has also focused on evaluating the outcomes of CC training in mental health. In an ethnographic study that evaluated the utility of a cultural sensitivity course for psychiatric residency training program, Willen (Willen, 2013; Willen et al., 2010) highlighted that trainees come in with differing levels of experience with diversity, which leads to varying responses to CC training. Conversations about cultural differences lead to intense emotions, and that such training assumes that the clinician is from the dominant mainstream culture, while the patient is culturally different. In a meta-analysis, Smith et al. (2006) found that multicultural education—when based in theory and research—was effective in increasing self-reported CC among mental health professionals, though effect sizes were small. The meta-analytic review indicated a range of duration of CC training from 2-week long workshops (40%) to semester-long courses or more involved training programs and concluded with the recommendation to continue the support for CC education. In a more recent review of 17 training outcome studies, CC training was shown to increase knowledge but findings were mixed for awareness, attitudes, and skills (Benuto et al., 2018). Overall, studies of CC in mental health in the US have primarily involved trainees and practitioners, with perspectives of educators missing in existing work.

### Cultural Competence in Healthcare in India

The literature on CC training in healthcare programs in the Global South is significantly limited. Our literature search revealed no published studies focusing on CC training in mental health in Africa, Asia, or Latin America, though a few published studies focus on CC in other health professions (i.e., medical, dental, nursing, or pharmacy programs). In one such study of nursing students across nine countries in Asia and Africa (including India), prior diversity training along with personal and professional diversity-related experiences (e.g., living in a culturally diverse environment, experiences of providing care to diverse patients) were positively associated with higher scores of CC (Cruz et al. (2018). There was a wide variation in CC total scores for students within each country and mean differences in CC across countries. The researchers did not address the possibility that CC may have different meanings across these countries based on dimensions of diversity that are most relevant in a given society.

In a descriptive article, Dangmei and Singh (2017) illustrate health disparities for caste oppressed individuals in India with poor health status, lower access to and consequently lower rates of utilization of health care services, and highlight the need for CC to reduce these disparities. The relevance of CC is also underscored in a study of dental, medical, nursing, and pharmacy students at a university in South India, in which medical students reported lower competence in meeting the needs of patients from diverse backgrounds, and in collecting information regarding health and patient beliefs and behaviors than nursing students (Balachandran et al., 2021). Research has been undertaken to establish reliability and validity of a self-report measure of CC for health care in a sample of students in *Ayurveda* (ancient Indian system of medical care), dentistry, homeopathy, medicine, and nursing (Balachandran et al., 2022). Although this scholarship highlights the relevance of CC for healthcare professionals in India, none of these articles focused on CC as it relates to mental health.

Graduate training in clinical psychology in India dates back to 1920s when one of the first courses in psychoanalysis was offered (Prabhu & Shankar, 2004), and by 1975, several universities offered courses in applied psychology (Prasadarao & Sudhir, 2001). As a country that was ruled by the British until 1947, India’s higher education system, including education in psychology, is highly influenced by Euro-American theories, research, and models of training (Prasadarao & Sudhir, 2001). Graduate curricula in clinical and counseling psychology in India have followed training models from the UK and USA with current emphasis on developing specific competencies (Srivastava, 2020). Alongside the developments of Euro-American systems of higher education and healthcare, the ancient Indian system of medical care, *Ayurveda*, which includes management of mental health, has continued to grow (Chakraborty, 2004). The mental health policy by the government of India calls for curricula that incorporate the local cultural elements (Ministry of Health & Welfare, 2014). However, no published research has examined CC training for mental health in India. Within the historical context of an ancient system of medicine and British rule, exploring training in CC in India can provide critical insights to training globally.

### The Aims of the Current Study

To address the gap of limited literature regarding CC training in mental health in different global contexts, our aim was to explore graduate students’, practicing clinicians’, and faculty members’ perspectives regarding the CC training they received (or provided, in case of faculty) and future training needs in the USA and India. We chose these two countries because they are highly diverse, though the most salient dimensions of diversity are different offering complementary contexts (Woodward & Saini, 2006) within which to examine CC training. For example, with more than 20 distinct languages spoken, a majority of the population residing in rural areas, socioeconomic differences evident in 32% of the population living below the poverty line, while over 6% has obtained a Bachelor’s degree or higher (Registrar General & Census Commissioner, India, 2011), and all of the major world religions represented with Hindus as a majority (Registrar General & Census Commissioner, India, 2011), diversity based on religion, ethnolinguistic groups, urban versus rural residence, and socioeconomic class are among the primary markers of diversity in India. In contrast, as a country of immigrants, in USA, 14% of its population is foreign-born (US Census Bureau, 2020a), 20% speaks a language other than English at home (US Census Bureau, 2020b) and 42.2% is racially or ethnically diverse (US Census Bureau, 2020c). Socio-economically, 11% of individuals live below the poverty line (US Census Bureau, 2020d), and 32% of the population has a Bachelor’s degree or higher (McElrath & Martin, 2021). The rich demographic diversity of these countries necessitate that mental health providers be attuned to differing cultural values, beliefs, practices, and lifestyles, providing a critical platform to examine CC training.

Using convergent parallel mixed-methods design with two parallel and independent strands of data collection and data analysis (focus groups and survey measures), we addressed the following research questions: (1) How do students, faculty, and practitioners describe their experience of CC training? (2) What challenges do they identify with CC training? And (3) What future training needs do they identify? Prior literature on CC in the US has primarily focused on trainees and practitioners, and thus, the inclusion of faculty as a key informant in our study provides a more comprehensive overview of CC training. Further, exploring these research questions in two highly diverse countries that offer complementary contexts may help demonstrate how specific components of training and challenges to such training may be contextually grounded.

## Method

### Participants

*Survey* We had completed surveys from a total of 450 individuals across geographic locations throughout India (graduate students = 327, faculty = 30, and practitioners = 40), and 350 in the USA from across 45 states (graduate students = 229, faculty = 80, and practitioners = 64). A majority of the participants were currently pursuing or had obtained graduate degrees in clinical or counseling psychology, with the remaining with specializations in school psychology, applied psychology, or other areas (see Table 1). A majority of faculty and practitioners in USA had obtained doctoral degrees. Faculty and practitioners in India had obtained either a doctoral degree, or a masters-level degree, which is consistent with practices in higher education and healthcare in India. A majority of survey participants identified as cisgender female and straight in both countries and as White in USA. Participants in each country represented the local religious diversity, with over half of the Indian survey participants reported following Hinduism.

**Table 1:** Survey sample demographics

	India			USA		
	Practitioner	Student	Faculty	Practitioner	Student	Faculty
Highest degree obtained						
PhD	15.8%	0.6%	41.4%	64.1%	3.1%	83.8%
PsyD	2.6%	0	0	21.9%	0.4%	7.5%
EdD	0	0	3.4%	0	0	1.3%
MPhil*	23.7%	0.3%	24.1%	–	–	–
Master’s degree	57.8%	45.6%	17.2%	9.4%	70.8%	0
Bachelor’s degree	0	52.6%	0	0	25.4%	0
Graduate degree specialty						
Applied	5.3%	20.2%	4%	0	0.9%	1.3%
Clinical	36.8%	53.4%	20%	68.8%	60.3%	59.5%
Counseling	34.2%	11.1%	32%	17.2%	25.3%	16.5%
School	2.6%	0.8%	0	6.3%	8.7%	7.6%
Other	21.1%	14.5%	44%	7.8%	4.8%	15.2%
Gender identity						
Cisgender female	85%	90.2%	86.2%	75%	82.5%	64.6%
Cisgender male	15%	9.2%	13.8%	25%	15.4%	35.4%
Non-binary/fluid	0	0.6%	0	0	2.2%	0
Sexual orientation						
Straight/heterosexual	88.5%	97.7%	100%	89.7%	83.1%	86.6%
Bisexual/demisexual/pansexual	11.5%	1.5%	0	5.2%	6%	9.2%
Gay/lesbian/queer	0	0.8%	0	5.2%	6.2%	7.5%
Asexual	0	0	0	0	1.4%	0
Race/ethnicity						
American Indian/Alaska Native	–	–	–	1.6%	0.4%	0%
Asian	–	–	–	14.3%	11.4%	7.6%
Black or African American	–	–	–	1.6%	5.7%	5.1%
Hispanic or Latinx	–	–	–	3.2%	8.8%	6.3%
White	–	–	–	73%	66.2%	75.9%
Multiracial	–	–	–	6.3%	7.5%	5.1%
Religious affiliation						
Atheist/Agnostic	5%	5%	6.9%	29%	36.7%	24%
Buddhist	2.5%	1.3%	0%	0%	0%	4%
Christian	12.5%	9.4%	13.7%	17.7%	19.7%	22.6%
Hindu	57.5%	57.7%	51.7%	4.8%	4.3%	1.3%
Jain	0%	0.3%	0	0	0	0
Jewish	0%	1.6%	0	4.8%	5.5%	10.7%
Muslim	5%	7.2%	10.3%	3.2%	0.9%	1.3
Sikh	0%	1.6%	0	1.6%	0	0%
Zoroastrian	2.5	0	0	1.6%	0%	0%
Other	15%	16%	17.2%	37.1%	32.6%	36%

Christian includes Baptist, Brethren, Lutheran, Methodist, Pentecostal, Protestant faiths

Degree specialty “other”: social or positive psychology primarily for faculty in India; pastoral counseling, mental health counseling, and marriage and family therapy for students and practitioners in both countries

*MPhil, or Master of philosophy is a degree a student can pursue after receiving MA or MSc in India, which provides a stepping stone to a doctoral degree

*Focus Groups* We conducted a total of 15 focus groups in India across four cities with 69 total participants, which included six with practitioners (*n* = 28), two with faculty (*n* = 10), and seven with students (*n* = 31). Each focus group had between 4 and 7 participants. The student and faculty focus groups included participants affiliated with master’s programs in clinical and counseling psychology (MSc and MPhil degrees), as well as a few that had PhD scholars. Participants predominantly self-identified as cisgender women (80.9%) and Hindu (66%). Practitioners included masters-level clinical or counseling psychologists, as well as those with a PhD. We completed a total of 10 focus groups in the US across four cities with 38 total participants, which included three with students (*n* = 12), two with faculty (*n* = 9), and five with practitioners (*n* = 17). Each group had between 3 and 5 participants. The student and faculty focus groups included participants affiliated with PhD or PsyD programs in clinical psychology. Practitioners included those with doctoral degrees and some masters-level clinical or counseling psychologists. Participants predominantly self-identified as cisgender women (76%) and White (65%).

### Procedure

*Survey* An email with an online survey link was sent to all APA-accredited masters and doctoral programs in clinical, counseling, and school psychology, and distributed to various national listservs for practitioners in USA (e.g., APA divisions’ listservs, regional psychological associations’ listservs). In India, online survey links were sent through email to psychology department chairs at universities and colleges across the country, as well as to listservs for Indian mental health practitioners. An average of 86% (range 75 to 96%) of those who clicked on the online survey link and read the consent screen across both countries completed the survey. When requested, paper copies of surveys were sent via postal mail, or if possible, a member of the research team brought paper copies of the surveys to a college campus and administered the surveys in-person to students and/or faculty. All survey data were collected in 2016–2017.

*Focus Groups* An electronic copy of the recruitment flyer for the focus group was sent to directors of clinical training of clinical or counseling psychology doctoral programs located within 100 miles of the primary author’s institution to recruit faculty and graduate students in USA. Electronic flyers were sent to a listserv of local clinical psychologists and printed copies were mailed to clinical psychologists in private practice, community mental health, and medical center settings located within 100 miles of the first author’s institution. Interested individuals were instructed to contact the first author to participate in a focus group. All focus groups in the US were conducted by the first author, an Indian American cisgender woman clinical psychologist or third author, a White American cisgender woman medical anthropologist. Focus groups were held at a university, medical center, mental health agency, or group practice setting.

In India, recruitment flyers were emailed or sent through postal mail to psychology department heads and psychologists in private practice, or community mental health non-government organizations or hospital settings. Psychology department heads and practicing psychologists were also contacted via phone to provide information about the study and solicit interest. Three research team members from the US (Indian American cisgender woman clinical psychologist, White American cisgender woman medical anthropologist, White American cisgender man clinical psychologist) conducted some of the initial focus groups along with three research team members from India (Indian cisgender man cultural psychologist and Indian cisgender women clinical psychologists), who then completed the remaining focus groups. Focus groups were held at a university, hospital, or community mental health clinic, and were conducted in 2016–2017.

Following conventional content analysis (Hsieh & Shannon, 2005), two members of the research team who are closely familiar with clinical training in both India and USA (an Indian American clinical psychologist and an Indian clinical psychology graduate student studying in USA) inductively develop a coding scheme. The data were coded by one of these researchers, and all the coding was reviewed by another who served as an auditor.

### Measures

*Survey* The survey included a demographics questionnaire with questions about personal demographics (age, gender, sexual orientation, race/ethnicity if relevant, first language if relevant, religious affiliation, parental education, state of current residence), as well as about participants’ education and training (highest degree obtained and year, specialty, current year of the training program for graduate students, training model of the program if relevant, licensure status if relevant).

To assess training in CC, we developed three questions: The first question assessed where in the curriculum CC training was occurring: A single course focused on CC, More than one course focused on CC, Non-CC focused course(s) with at least one full unit/class/section dedicated to CC, Infusion of CC throughout courses, Clinical experience with clients of culturally diverse backgrounds, Research experience with culturally diverse participants, Research about CC, advisement/mentorship by a faculty member whose background is culturally different from your own, or none of the above. Participants were asked to check all that they received as a part of their graduate clinical training. The second and third questions assessed satisfaction with CC training with respect to various dimensions of diversity (age groups, country of origin, education, gender, life style preferences, physical ability, race/ethnicity, refugee status, religion, sexual orientation, and socioeconomic status), and the desire for additional training. For satisfaction, participants were provided with an option 0 = did not receive training in this area, along with a rating scale 1 (Not at all)—5 (very satisfied). For additional training, participants responded on a 5-point scale: 1 (Not at all)—5 (very important).

*Focus Group Interview Protocol* The protocol that we developed included five main questions. The initial two questions were broad and sought to gather information more generally about the participants’ training: (1) Thinking about the training you received, in what areas do you wish you had more training to be prepared for the kind of work you do?, (2) What are/were the strongest aspects of your training? Next, participants were asked questions specifically focused on CC: (3) Are you familiar with the term CC? (4) What kind of training have you received/are receiving in CC? (5) What kind of additional training in CC would you like? For faculty participants, two additional questions were asked: (6) Thinking about the clinical courses you teach, how do you integrate CC in your course?, and (7) What challenges do you experience in trying to integrate CC in your courses?

## Results

### Quantitative (Survey) Findings

**Fig. 1:**
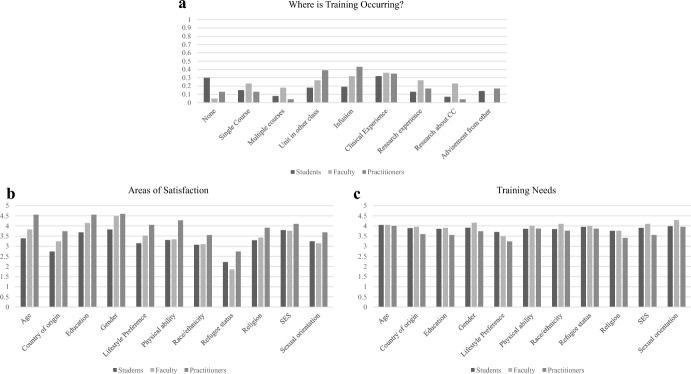
**a**–**c** Indian participants’ ratings of where CC training is occurring, areas of satisfaction, and further training needs

**Fig. 2:**
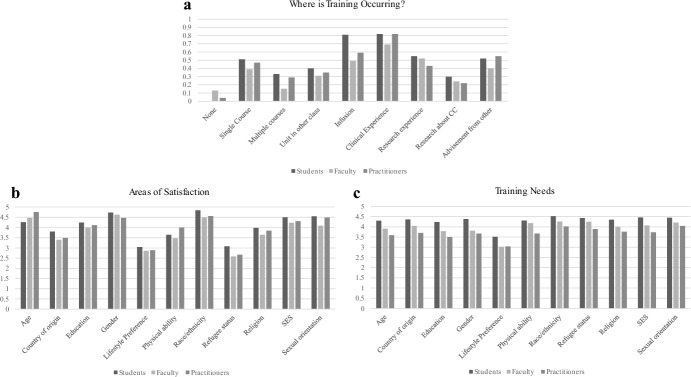
**a**–**c** US participants’ ratings of where CC training is occurring, areas of satisfaction, and further training needs

*Where in the Curriculum is CC Training Occurring?* In India, 30% of students reported receiving no formal CC training, while 32% reported obtaining it through clinical experiences. Faculty reported obtaining CC training primarily through research (36%) and clinical (32%) experiences. In contrast, practitioners reported CC training through coursework, with 39% reporting a CC unit in other classes, and 43% reporting infusion throughout the curriculum (See Fig. 1a). In the USA, students reported CC training through infusion across the graduate curriculum (81%) and through clinical experiences (82%), while faculty reported obtaining CC training primarily through research (69%) and clinical (52%) experiences. Similar to students, practitioners reported CC training through infusion across the graduate curriculum (43%) and through clinical experiences (81%) (See Fig. 2a).

*Areas of Satisfaction With CC Training* Across participants in India, the area of highest satisfaction with respect to CC training was gender identity. In addition, students rated SES, faculty rated education, and practitioners rated age and education as areas where they were satisfied with their training (see Fig. 1b for mean ratings across type of respondent). For participants in USA, the area of highest satisfaction with respect to CC training was race/ethnicity across respondents. In addition, students and faculty rated gender, and practitioners rated age as an area where they were satisfied with their training (see Fig. 2b).

*Areas Where Additional CC Training is Needed* Across participants in India, the area of highest need for additional CC training was sexual orientation. In addition, students and practitioners rated age, and faculty rated gender, as an area where they needed additional training (see Fig. 1c for mean ratings across type of respondent). In the US, the area of highest need for further training across respondents was race and ethnicity. In addition, students rated SES, faculty rated refugee status, and practitioners rated sexual orientation as an area where they needed additional training (see Fig. 2c).

### Qualitative Findings

**Fig. 3:**
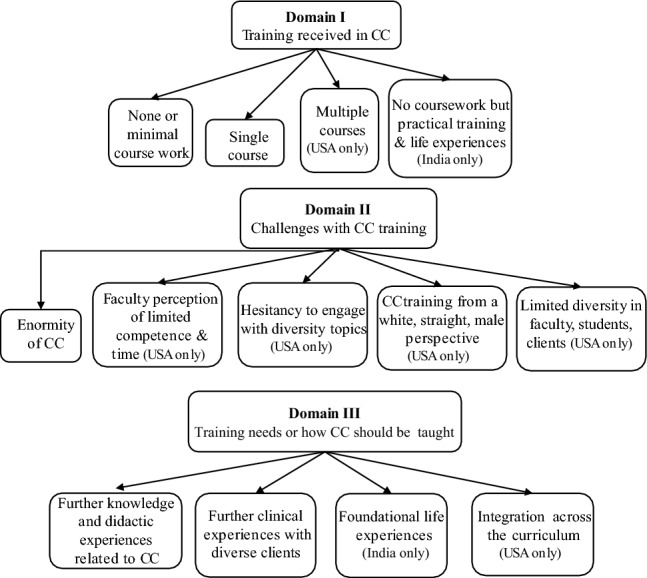
Thematic map of coding categories

A total of 13 themes were identified from the focus groups that were organized into three domains: (a) training received in CC, (b) challenges associated with CC training, and (c) training needs or how CC should be taught. Some themes were coded in focus groups conducted in both countries, and others were coded in focus groups from only India or only USA. See Fig. 3 for an overall thematic map.

#### Training Received in CC

Participants’ responses to the training they received in CC in their graduate programs were coded into the four categories: none or minimal coursework in CC (both countries), single course in CC (both countries), multiple courses addressing CC (only in USA), and no coursework but practical clinical experiences and life experiences (only in India).

*None or Minimal Coursework in CC (Coded in Both Countries)* Some participants, including faculty, graduate students, and practitioners in India and some practitioners in the USA described having received minimal coursework in CC. Specifically, some faculty members in India described that although they do not cover CC in coursework formally, faculty and students alike have a lot of experience interacting with individuals who have different social identities than their own. Graduate students and practitioners in India described the importance of having formal training in CC to complement their experiences and some shared that they had discussions about the sociocultural backgrounds of their clients in a few of their classes. A faculty member in India said, “At this point, we can say it because we have loads of experience behind us… I do not think there is any program or structured way of teaching such a thing… So, we learn by ourselves.” Similarly, another graduate student in India spoke about the need to make up for the lack of formal training on CC: “Since we have no training to begin with then I think to basic, basic trainings of about how to be with… Just more knowledge about what are the different kind of…What are the different backgrounds that people can come from? How do we deal with them?” A practitioner in India added,

If I meet a client who is from Northeast, okay? Am I competent enough to understand what the child is coming from? The background of the child, the culture of the child… So, we have discussed about this, but not much. It was just two or three classes.

Practitioners in the USA who received their graduate training in later 1980s and early 1990s shared that they their training focused on what were considered to be core clinical topics at the time with limited focus on applicability of those topics for diverse groups. For example, a practitioner in the US stated, “Well I graduated in 1987 um and so my program… The only course I can even remember touching on ethnicity was the Family Systems Course and it was one text really.” Another practitioner elaborated, “I imagine that today there’s more, a lot more uh coursework in graduate schools than there was in the late nineteen eighties and early nineteen nineties.” A third practitioner said, “I feel like my training experience helped me to develop this really solid core of understanding how human change occurs and what the processes are. It didn’t apply it to this broad and important range of context of culture. Not very much.”

*Single Course in CC (Coded in Both Countries)* Some participants in both countries described taking a single course on CC or multicultural psychology through their training. For example, some graduate students in India described a course in social psychology that included content related to cultural diversity.

But we do have a subject. We do have the subject on social culture so Social Psychology. So, we do have a subject but that teaches a lot about myself and also other cultures. But then again India is a place where you cannot I think cover all the cultures and all the and you know other of gestures that they make. Everything. So, it’s all based on the practicum and reading on cultural issues that we need to work on. That [we] ourselves need to work on. A lot of thinking is required. And interacting, being open to interact with anyone you think or anyone who is available.

Similarly, some graduate students in USA described having one diversity course in their curriculum, while underscoring the need for integration throughout the curriculum:

Well, we have that Diversity class [chuckles]. And then, however much it’s integrated into the other courses that we have to take um, I think I’ve been lucky in that the settings that I’ve been in have exposed me to a lot of different things.

Another graduate student in the United States described a similar experience:

That one course was really great, …” But elsewhere in the curriculum “So when we’re having conversations about diagnoses and the response is ‘Oh, well, you’ll talk about the in [multi] cultural class” or “You’ll get that on traineeship” to me that’s frustrating because embedded in that is, ‘Well this is the set of diagnostic criteria for regular people and then you’ll talk about nonwhite people later’…there’s a privilege inherent in that and it’s frustrating.

*Multiple Courses Addressing CC (Coded Only in USA)* Some graduate students, faculty, and practitioners in USA described that their training programs incorporated diversity throughout the curriculum with some programs having an introductory course on multicultural psychology that includes experiential and self-reflective components followed by discussions of social identities in all clinical courses. Other programs had a curriculum committee that facilitated integration of diversity throughout the curriculum. Some graduate students commented on the role of the instructor and individual differences in the instructors such that some faculty had specific days or weeks designated for diversity in their course syllabi, whereas others were able to integrate diversity throughout the course. For example, a practitioner in the US stated, “I graduated in 1988, one year after… at the University [name of school] and there was almost a running joke in our program because every single course integrated cultural diversity, cultural sensitivity and cultural awareness.” Upon being asked how CC is being infused in the program, a faculty member described,

Every student has a first-year course that is a very broad introduction into multiculturalism and diversity. That’s sort of intended to um think about uh the role of identity and status across all the different [pause] you know, race, class, gender, age, religion, and um socio-sexual orientation etc. just on and on. And then students are invited into a fair amount of experiential reflection in that class. They’re expected to do some kind of project where they identify some area of discomfort and they’re supposed to go immerse themselves in some way in that community. Students would go to an African American church or go to a gay bar or go take a walk in a neighborhood uh where people are impoverished or something, and there are various projects that people do. Then in addition to that in every course and the curriculum committee has monitored this and we do it periodically… People have you know readings that are reflecting of current clients with respect to different groups.

Similarly, another student in the US spoke about the different courses incorporating CC, while also acknowledging the role of the instructor.

There [are] some professors who model that. It’s not like there’s -- it’s a requirement that you do that. There’s some diversity once every quarter and you can tell in some courses it’s just like, “Oh, this is a diversity day. This is what we’re going to talk about as we check that box.” Other professors integrate it more seamlessly into what we’re actually talking about. Um and I think there are other courses that allow that I guess more easily. We take a course called Professional Development but it’s basically like a semester on therapy. Then a semester on Assessment and I think since that course ends up smaller. It lends itself to more discussions and it’s easier to introduce more diversity into that. And so the professor I had for that I think did a good job doing that and it felt more comfortable than in other courses.

*No Coursework but Practical Training and Life Experiences (Coded Only in India)* Participants in India described that although the coursework on CC was limited in their training, there were plenty of clinical training experiences that facilitated the development of CC. For example, graduate students described relying on the knowledge of the staff at their traineeship sites to learn how to engage in culturally competent clinical practice with individuals who follow different religions, and who come from different parts of India. They reported learning clinical theory in their coursework and its application to diverse populations at various traineeship sites. As one graduate student described,

Somehow, we developed it. When you go to the hospitals, especially government hospitals. It’s not written in the papers. It’s based on the experience of the staff … because in there is the culture of the country. Respect, counseling… in it… (Interviewer clarifying: “So it seems like this was not part of any theory that you learned but more by experience you figured out that this was necessary.”) Yeah. Yeah. So then, it would not be possible to work with Indian settings. So, we have to understand their behavior first… Based on that we have to put theory into practice. It has to be in that context. We cannot directly apply theory.

A practitioner also elaborated on hands-on training during clinical practice:

Cultural context is something that we are not trained in during our graduate level training. Many a times when we’re in private practice or in a clinic or hospital, you know, we get people coming from different parts of India and within that area there are different states – indigenous individuals coming from different states into our practice.

Participants also described the rich life experiences of growing up in diverse communities that provided ample opportunities to develop CC. In particular, graduate students described how their life experiences were helpful in filling the gap of not having formal coursework on CC. As one student illustrated,

I think it’s just like Indian University. It’s just common sense. Because we have grown up knowing that there is so much that is mysterious and we’ve grown up acknowledging those differences between people. So even though we don’t like formally they haven’t taught us to do it – I think it is just there. Yeah, we just know. It is not like we don’t talk about it in our classes. Obviously, we talk about during our presentations. People raise issues that okay is this a cultural practice or is this normal? What is it for clients? I think it is just an understanding that exists.

#### Challenges Associated with CC Training

Five major challenges were identified including enormity of CC (both countries), faculty perception of their own limited competence and limited time (only in USA), individual difficulty in engaging with diversity topics (only in USA), CC training from a white, straight, male perspective (only in USA), and limited diversity in faculty, students, and clients (only in USA).

*Enormity of CC (Coded in Both Countries)* Across focus groups in both countries, participants discussed that knowledge and skills related to CC are vast and voiced concerns about familiarizing themselves with a range of cultural groups and clinical presentations. Graduate students in India referenced the vast differences in languages, food, clothing, and lifestyles across the country and that it would not be possible to learn about all of these differing presentations through one’s graduate training. For example,

So, if you are working like in your native place, you can still maybe you know use language to your advantage, or you know how the people live there. You realize that. You know what they eat, their way of talking, their way of dressing so you can just use that to your advantage. But if you are in some other state, other than your own native state... Even in your own state, if you go to some other city, it is very different. So even if it’s included as a part of our training, how much will they teach us? I don’t think all the years of our lives would be enough to learn about everything!

Graduate students in USA shared that no single course or even an entire graduate curriculum can prepare one to competently provide clinical services to clients with all possible intersecting social identities. As one student articulated,

How are you supposed to be able to relate to so many different types of cultures? How do you understand them, you don’t know their history, you don’t know their lives, you don’t know what things mean for them, and it is so diverse, it is a challenge.

Participants across multiple focus groups discussed CC as a life-long process, that it is not something someone achieves, but is an ongoing process of constant awareness of overlapping and dynamic identities that impact clients. As one practitioner in the US explained,

To me, it’s an aspirational thing. I don’t think it’s like I’m culturally competent. It’s something you continue to aspire to and to me there are times, like “Hey, you can feel a sense of cultural competence” and there’s times when like “Hey, I’m not being culturally competent.

*Faculty Perception of Their own Limited Competence and Limited Time (Coded Only in USA)* Faculty teaching in graduate programs in the USA shared that they may not consider themselves as competent and qualified to teach about CC because the training they received in CC in their graduate programs was highly limited. Some faculty members talked about not having the foundational knowledge necessary to teach CC. Multiple times faculty lamented that they did not have the kind of in-depth professional training they felt they needed to feel confident in the training they were providing students with, and that time to devote to their own training was structurally unsupported within the university system. For example, one faculty member stated, “I think that not having any foundation, like zero foundation, and then going into this [cultural competence] very high level [of training]….I think this is reflective of other faculty, I feel very insecure about this.” Another faculty member shared a similar experience: “My training was nowhere near as good as I think their [current graduate students] training is in cultural competence. But then I’m expected to kind of be the instructor in this area.”

In addition to limited competence, faculty also shared that there was insufficient time to cover CC in their courses. In particular, they discussed balancing different priorities and finding time in a semester-long course that is not explicitly about diversity (e.g., courses such as psychopathology, assessment, intervention) to cover content related to diversity.

I just started teaching, I’m in the middle of teaching my class right now which is a relatively new one. But just spending a lot of time trying to think about all the different groups you want your students to consider as they’re learning the material and how to [pause] infuse that in [pause] and how much time you have to do that. And it’s like teaching the topic that you have and how do you bring in [pause] how to apply that to diverse groups [pause] um with enough range and still having enough time to delve into -- like I’m teaching psychopathology [pause], learning about all of that and applying the different groups that they might see. Um I think of just having enough time to do that and make sense without overwhelming them with readings. Um just enough to be deep enough that they [pause] understand how to do that and take it and apply it to different groups that they might have to work with.

*Hesitancy to Engage with Diversity Topics (Coded Only in USA)* Graduate students discussed faculty members’ and their fellow students’ lack of willingness to discuss diversity-related matters and difficulty acknowledging and working through biases that are embedded in the discipline. In particular, some students discussed faculty members’ difficulty or unwillingness to acknowledge theories and research that represent the views of only White individuals. From students’ perspectives, when they brought up racist and sexist underpinnings of the knowledgebase in psychology in their classes, the faculty instructors seemed to “gloss over” or “move on” rather than engaging with and encouraging these conversations. There were also references to bigger class size as a potential barrier interfering with student engagement and willingness due to concerns around lack of privacy. Some students also brought up their supervisors’ unwillingness to engage with diverse perspectives in clinical supervision, where supervisors were perceived as “skirting the topic” or not engaging in a way that felt “authentic.” One graduate student stated,

And certainly, in Theories of Personality class the entire [group of theorists] is white men and I think that if you’re going to be teaching that, whatever… There were things like that brought up. “Oh well consider the time they were writing,” and I think that we need to move beyond that.” You can’t make this profession more welcoming and diverse and to learn how to work with people who are different than the majority culture if you’re just going to be like let’s just gloss over that.

Another graduate student provided their perspective on the same issue:

Also, it just felt like “consider the time that it was written”. Like this wasn’t an unusual thing to say so let’s just move on from that and not discuss how like this is now, like you know a racist view to have or like a sexist way to treat people. So. It just felt like “I don’t want to talk about it” when you say that.”

A few graduate students highlighted this issue within clinical supervision:

I didn’t mean to say that because they are white, they couldn’t help but it seemed like they didn’t want to talk about it. Um and I’ve had like work with other people who were like we were willing to talk about it. It’s like just my individual supervisor, those ones that I’ve had over the years, it doesn’t feel like it’s been good -- and helpful -- that part of it.” (Interviewer: “So you feel like you bring up issues but they don’t get discussed?”) “Yeah, it’s kind of like they get dropped pretty quickly. They find some way to skirt the topic.

Um in terms of whenever I talk about racial interplays with clients, they’ve -- it always feels like they’re coming from the outsider’s perspective in trying to um see “Okay, I as a white person” trying to enter into this relationship with say a Latina or a black person and it’s just -- it doesn’t feel authentic, it doesn’t feel organic. It feels um artificial because it’s coming from the outside.

Faculty instructors also spoke about students’ difficulty with engaging with topics related to diversity, and individual differences in students’ own privilege, their training needs, and their preparedness to engage in reflections and conversations about diversity. As one faculty member explained,

I think one of the biggest challenges is trying to meet the individual training needs of students when they’re in such different places. Um and some are so ready to kind of [pause] you know, have a big opening up of their [chuckles] experience and um and realize like -- especially -- again you know especially students who come from a certain amount of privilege [pause] just realize how much they didn’t ever think about that very topic and the consequences of that.

A student highlighted the challenges their peers experienced:

Just from like the first-year experience I think it might be helpful to have the diversity class be smaller or at least at times meet in smaller groups. I think a major obstacle to having really good discussions in that class for at least my cohort was we were all there, so if you said something that you felt like people weren’t going to agree with there was that like “Oh gosh, everyone’s going to know” kind of feeling and I think that really prevented some of the discussions that we could have had.”

Some practitioners referenced “hostility” and that White trainees felt that they “got beat up” for their privilege:

“I can remember when it was first getting really big, everywhere you went they were offering this diversity training and cultural competency training and there was hostility and there was you know people resistant. And they didn’t want to talk about it because you know if you’re white you got beat up, a lot of people felt and if you, you know, were told how terrible you were and, you know, how you needed to recognize your privilege which, yeah we do but again there’s approaches.

*CC Training from a White, Straight, (cis) Male Perspective (Coded Only in USA)* Graduate students and practitioners shared the view that CC was taught from specific perspective such that (a) there were monolithic portrayals of cultural groups, (b) CC was taught with the assumption that the trainee or the therapist was White and the client was a person of color, and (c) uncertainty or lack of recognition of white, straight, male as cultural identities. In particular, practitioners who were trained in 1990s shared that when diversity was included in their curriculum, there were often monolithic descriptions of various communities of color in their textbooks without recognition of individual differences within a community or of intersectionality. A practitioner narrated,

All the early training I did in cultural… it was like okay, well this is half a seminar or a workshop on culture, on Hispanic culture as if there are these monolithic things that all black people share these characteristics. Um you know and that’s – a lot of the early training really re-enforced stereotypes and really angered a lot of people.

Another practitioner further highlighted this issue: “Training in the 1990s consisted of four groups Asian American, All American, African American, and Native American and Latino/Latina, that’s it, that was the diversity [and everyone was presumed to] fit into very clean boxes.”

In addition, graduate students described a second problem with contemporary CC training, that the training did not address the needs of trainees of color. In particular, graduate students of color felt that they were not gaining much from what they were learning because CC training assumed that the trainees were White and there was no discussion of the experiences of trainees of color leaving trainees of color to feel “left out.” As one graduate student discussed,

Um I think just diversity training in general is a weakness of our program. And I think like as a black woman I’ve often felt like the way they teach us in from the perspective of like a white dominant culture therapist and like how you would interact with all these different people instead of discussing how um…I’ve often felt like left out in discussions that we have um because it seems like they only want to help – because it is a predominantly white program – and so it’s often geared toward helping other people get through that. Which isn’t bad for them, but I mean it makes me feel weird and kind of unsure where I can contribute to this discussion. And what I can gain from it.

Similarly, another graduate student said,

And I also think that um also being a minority male adds to that kind of whatever stereotypes and beliefs that they might have uh towards uh Latino men – plays out in some way, shape or form. And then I get [pause] I really [hesitates] didn’t take into consideration as much as the past two years now. So that’s been an area that I would have liked to gain a little bit more of, of the difference [pause] – between them.

A third and related problem that was identified with CC training was the difficulty or lack of acknowledgment of whiteness as a culture and white as a social identity. Some practitioners trained in the 1990s talked about beginning to engage in the discussion of whiteness as cultural in their training programs, though current graduate students of color felt that their fellow white trainees did not see themselves as cultural beings. Students or faculty with racially or sexually minoritized identities were the only ones perceived as “multicultural.” For example, one practitioner stated,

We did talk about whiteness. I mean we started to talk about it…there was a piece of knowledge that you know whiteness was its own identity and that needed to be looked at, but [the conclusion was] ‘we’re not quite sure how to do that yet.’

Some graduate students also felt that this lack of recognition of white as a social identity resulted in discussions of a majority of psychological literature that is based on White Americans as “normal psychology” and the emerging scientific knowledgebase about people of color as multicultural psychology. A graduate student of color (referring to a conversation with a peer) stated, “‘You’re so lucky that you have culture. I don’t because I’m a white girl.’ And I’m like “I meet your culture every day.” Another graduate student also highlighted this point: “Everybody else is, you know, like all of these straight white people who, it doesn’t matter because we know the ‘normal psychology’ and then you’re [referencing the ‘multicultural faculty’] there to upload the multicultural competence and teach us about non-white, straight people.”

*Limited Diversity in Faculty, Students, and Clients (Coded Only in USA)* The need to diversify faculty and graduate students was brought a number of times. For example, some graduate students of color mentioned not having faculty instructors or supervisors who share their racial identity, and how little the faculty composition has changed over the years. One graduate student of color stated, “I should also say that none of my supervisors have been of the same race as me, so I think that also makes it peculiar to me.” Another expanded,

Um my grandfather is -- uh well, he’s still alive but he retired -- he was [laughs] a [person of color] psychologist and but the things -- he told me things about like his training and what it was like for him. And while things are definitely better for me, this -- some things are still kind of the same. He was in grad school almost 60 years ago, so it’s like -- I think we like to believe that we’ve come so far, but I’m not sure that we have. Um and going to conferences and like seeing the people who are doing most of the research, it’s mostly like white dominant cultured people. So, I think just recruiting more diverse psychologists would lend itself to having a more diverse field like in what we actually do and the things we send out.

Other graduate students described the benefits of a diverse community of students and faculty for providing deeper training in CC where a variety of different life experiences can be discussed and there are opportunities to interact with individuals with different social identities. As one graduate student expressed, “Well I think the student body itself could be more diverse. I think that would help.” Another Graduate student expanded,

I also think it would be nice if the diversity came in like the person teaching it because much of our diversity training especially as first year came from a white woman and it would have been nice to have seen um diversity spoken of by people of many, many diverse groups.

A practitioner also shared a similar view:

Graduate training needs to do a better job of recruiting students who are more diverse, too, so that we have that – and faculty – so that we have those opportunities to interact with people and to know and appreciate the value of differences.

#### Training Needs or How CC Should be Taught

Participants shared the following as important in further developing CC: further knowledge and didactic activities related to CC (both countries), further clinical experiences with diverse clients (both countries), foundational life experiences (India only), and integration across the curriculum (USA only).

*Further Knowledge and Didactic Activities Related to CC (Coded in Both Countries)* Participants in both countries shared the view that it was important to have theoretical knowledge related to diverse identities in graduate training, as well as in continuing education initiatives. Graduate students and practitioners in India described why CC is critical to work with clients from different subcultures of India and the need to have CC be a part of formal coursework. As one student stated, “I did not find that in India the syllabus of the universities. It has to be taught. So that they will get toward religions and they will not be biased when they are talking to the patients.” Similarly, a practitioner shared,

I think, you know taking from everybody that’s already spoken to it, from a culture specific and how to practice in different cultural contexts. We have around so many states in India and each state has a different culture, within in each state there are different subcultures. So, I think that this needs cultural competence and important amount of training that we should have.

Graduate students in USA identified specific areas where they felt further knowledge was needed (e.g., religious diversity), and the need for self-learning and seeking out opportunities to gain further knowledge, for example, through professional conferences. One student suggested,

I don’t think we had enough training in religious diversity because it’s you know easy to underestimate how many religious people are around and how many of them are our clients. And for me, being very skeptical about religion primarily and Christianity in particular it’s you know challenging and I think it’s important to learn more about different religious beliefs… I think that definitely I would personally benefit from more training.

Another graduate student discussed seeking out further knowledge:

I guess just continued trainings that doing research, going to conferences. Like when I go to conferences, I’m usually drawn to the presentations that have some sort of diversity thing in the topic. So, I guess just seeking it out on my own when no classes.

*Further Clinical Experiences with Diverse Clients (Coded in Both Countries)* Participants in both countries described the importance of continued clinical practice experiences with clients with diverse identities as essential to their building CC. For some participants, this entailed continued exposure to diverse groups, whereas for others, they sought targeted experience with individuals with specific social identities. A graduate student in India stated,

Rather than training it’s more like the practicum, more practicum because that’s the only way you can actually understand it. You can’t really get it through like a class or a paper or anything. So more practical exposure.

A practitioner in USA had a similar perspective:

What they did have in my school which is a good thing to do is if not mandate, certainly strongly encourage graduate students to work with populations that they’re uncomfortable with or where they feel initially, they might be uncomfortable with them but maybe they could grow into it… I am so glad that I worked at the pediatric hospital and at the university because if this were my first job, I wouldn’t know so much of the world… Because a very small percentage of the world comes into this office here. So, I think getting a diverse experience, giving you broad and deep but certainly a broad experience is really – it’s helpful…The more I learn about other people, I also learn about myself and that’s important. A lot of different experiences in graduate school during practicums here and there to figure out what you want to be when you grow up or what kind of psychology you want to do…

*Foundational Life Experiences (Coded Only in India)* Participants in India discussed the importance of foundational life experiences that are needed to develop CC, especially exposure from a young age. Specifically, some students felt that teaching CC at the graduate level might be too late because an individual’s values, beliefs, and worldviews are already shaped. Teaching children to be accepting of and open to different beliefs and lifestyles was considered to be a better approach. As one student stated, “I think it should be taught at a very young age because if you teach at a post graduate level, I don’t know… You are – you are – I think you are already formed.” Another student elaborated, “It has to be part of the earliest training. Yeah, because your culture – you already have a culture like when you [are] 22–23. It will be very difficult for you [to] pick up things like religion [and] culture when you are really young because most of the things you pick from the society when you are really young either from your parents or...”

Other students discussed the importance of life experiences in developing CC as an ongoing process, and elaborated on the need to continue to learn about different cultures and subcultures and this learning should be considered just as important as core clinical subjects such as assessment and psychopathology. One graduate student narrated,

We just need to be very accepting of the wide range of cultures that they have because I think like she said it’s no way possible for one individual to understand thousands of different cultures that there is in this country. I think we need to be accepting of that and that itself will help you understand people, and that should start early.

Another graduate student described,

So to just learn about that culture would be as important as learning one subject itself. Because even the subtlety, as westernized as they are [referring to urban, middle-class] there are still several things that their culture carries that is in each of us. So to know those things, so to spend extra time to learn as much as we can about these cultures, as much -- the important we give to subjects should be as equal as the importance of the gift of learning about the different cultures.

*Integration Across the Curriculum (Coded Only in USA)* Across multiple focus groups, participants in USA shared that CC training should be integrated across the graduate curriculum. Students and practitioners lamented that CC training couldn’t be limited or “partitioned off and treated as if it [it] can be separated’ from other aspects of training.”” Integration across the curriculum was also seen as evidence of commitment to valuing CC by all members of the training program. As one graduate student reflected,

It would have been nice to feel like there was more of a commitment to valuing cultural competence from all faculty….I think that there wasn’t a commitment or even really encouragement to engage in those types of conversations outside of the ‘Cultural’ course.

Another student expressed,

It would be nice if this could be integrated more because it’s like “Oh, we have a Diversity Class. We’re all set.” But I notice – and some of our classes our professors are very good about trying to talk about these things, but in some of them if you brought up a diversity issue [it] kind of just got shut down.

A third student recommended,

Make it so it wasn’t just [giggle] well we’re going to talk about black people today. No. You might bring an article written by someone who’s diverse or something like that and it’s just something every day not just on that one particular day.

Finally, another graduate student suggested,

I think just greater depth in the training that we do get because I think they try to be really broad in teaching you about all these different kinds of groups but then like you don’t have any meaningful discussions about it and I think certain people who maybe come from more like sheltered backgrounds don’t get as much exposure as – when they don’t actually talk about what it means to work with someone who is different.

## Discussion

Our findings from the survey and focus groups converge in highlighting the unique dimensions of diversity and cultural, educational, and healthcare contexts in each country that likely shape how and where CC training takes place. Participants in India described a practical emphasis to their CC training (e.g., learning about CC through life experiences and clinical practice experiences) more so than through coursework, whereas participants in USA described engaging with varying levels of coursework-related CC while identifying limited perspectives incorporated in this training. Strengths related to CC training in each country are mutually informative, and based on these findings, we provide recommendations for training programs in both countries.

### Cultural Competence Training in India

Across the survey and focus groups, some participants in India reported that they did not receive formal coursework in CC through their degree programs. For example, 30% of graduate student survey respondents in India reported receiving no formal training in CC, whereas faculty reported learning CC primarily through research and clinical experiences. Focus group data provided further insight into where and how CC training may be taking place. Although training programs may not include coursework specifically targeting CC, or use the term CC, there seemed to be opportunities to learn about how to engage in clinical practice with diverse clients. In particular, participants highlighted the role of life experiences that were formative in learning about diversity, and clinical experiences that built on those life experiences in facilitating CC. Participants referenced growing up in religiously, linguistically, culturally, and socio-economically diverse communities that helped them learn about diverse viewpoints and experiences. Some participants also referenced class presentations and discussions related to diversity, and others discussed clinical training experiences including learning from the wealth of professional experiences of staff members at the clinics and hospitals. In discussing future training needs, participants highlighted the importance of being open and accepting of differences, having theoretical knowledge related to diversity in their graduate coursework, exposure to diversity early on in one’s life and continued life experiences, and the need to spend extra time to learn about the client’s subculture, overall highlighting CC as a life-long process.

Clearly, trainees in India benefit from the diverse perspectives and lifestyles they are exposed to while growing up in communities that are religiously, ethnolinguistically, and socio-economically diverse. The clinical training experiences through internships also provide rich opportunities to interact with clients who come from a variety of different subcultures of India and to learn from staff at these training sites who have a wealth of experience in working with these diverse clients. It is important to recognize that although rich life experiences and clinical practice experiences provide a solid foundation, they may not be sufficient in developing CC. Consistent with our findings highlighting the need for theoretical foundation for CC in coursework, nursing students in India also reported less familiarity with theoretical bases of CC due to this topic not being included in their baccalaureate nursing programs (Cruz et al., 2018). Similarly, a study of clinical psychology trainees from India that did not focus on CC also described the strength of practical training and the need for focusing more on theory and research (Malik & Grover, 2014). A strong theoretical foundation in CC is critical in helping students to conceptualize their experiences with diverse clients, provide the vocabulary to discuss those experiences, reflect on their own values and how these might contribute to their clinical work, and help develop the skills to provide effective mental health services to diverse clients.

*Recommendations* Graduate training programs in clinical and counseling psychology in India may consider a more explicit focus on CC that is integrated throughout the curriculum including courses on assessment, psychopathology, diagnosis, and intervention. Coursework may address the theoretical foundation of CC or cultural humility, cultural foundations of human behavior including mental health and illness, and intersecting social identities in India based on age, education, gender language, lifestyle, religion, sexual orientation, social class, and urban versus rural residence that contribute to varying levels of privilege and marginalization. Survey responses indicated that although participants were satisfied with the training they received pertaining to age, gender, education, and social class, learning about diverse experiences based on sexual orientation and age was identified as the highest need for further training. Thus, graduate training programs may consider including scholarly articles pertaining to the development of sexual orientation, experiences of LGBTQ+ individuals within communities in India (including trans, non-binary, and third gender communities), as well as experiences of individuals across cohorts and across the life-span.

Scholarly articles that describe relevant theoretical frameworks (i.e., relational ecological model within India; Aggarwal et al., 2022), local idioms of distress, explanatory models of mental illness, and phenomenological experiences of psychiatric disorders within communities in India (Nichter, 2010), as well as indigenous perspectives on healing (e.g., Varghese et al., 2011) may be particularly relevant to include. Written assignments and structured class discussions that facilitate students’ deeper engagement with the scholarly articles, and structured opportunities that enable students to reflect on their own rich life experiences of growing up in diverse communities and identifying their own biases may be helpful. Further, as identified in a study that included mental health professionals from USA, India, and other countries, role-play and video demonstration may be effective methods of teaching CC (Aggarwal et al., 2016a, 2016b). When textbooks and peer-reviewed journal articles that are based primarily on research conducted with samples from the Global North are included, explicit discussion of the applicability of those theories, constructs, and evidence for communities in India is warranted. For example, the discussion could include the relevance of standardized tests originating from the Global North for various subgroups within India, along with how to best adapt empirically supported interventions originating from the Global North to be responsive to Indian clients’ sociocultural identities.

### Cultural Competence Training in USA

In early 1990s, a majority of counseling psychology programs in the US included at least one course on CC, and more than half had units on CC across multiple courses (Hills & Strozier, 1992). In comparison, two decades later, over 80% of US students responding to our survey indicated infusion across curriculum, demonstrating that CC is being integrated more in health service psychology curricula. However, the extent to which infusion is occurring seems variable as described by our focus group participants: some courses have been weaving in diversity throughout the syllabus, whereas others rely on distinct units or days dedicated to diversity. This variability is consistent with other recent research (Gregus et al., 2020) that suggests infusion as a growth area of CC training in health service psychology in the US.

A major challenge with CC training identified was that the training was geared toward a white, straight, cisgender male clinician working with clients with minoritized identities and related issues of monolithic portrayals of various groups. This limitation of CC training is consistent with the anthropological critiques of models of CC—viewing culture as static that leads to stereotypic views of racial ethnic groups without consideration of diversity of lived experiences within groups based on intersecting identities (Carpenter-Song et al., 2007; DelVecchio Good & Hannah, 2015; Kirmayer, 2012a). This limitation identified by our US participants is also consistent with the perceptions of clinical psychology trainees in Australia who reported a western bias in their CC training (Geerlings et al., 2014). Willen (2013) identified this limitation as well for US psychiatry residents through the “paradigm of ‘mainstream’ clinician vs. ‘other’ patient” (p. 249) that misses the heterogeneity among the residents being trained. Similar to our psychology graduate students, psychiatry residents in Willen’s ethnographic work (Willen, 2013; Willen et al., 2010) also discuss faculty and students’ hesitancy in engaging with diversity topics, and the powerful feelings of anxiety, frustration, and disappointment that are generated by these discussions. Similar to the psychology faculty in our study describing their own limited competence and insufficient time as challenges, the instructor in Willen’s (2013) study also described time constraints as a barrier to incorporating CC earlier in psychiatric residency. Finally, the challenge of limited diversity within the faculty and student body and the need to diversify graduate programs is critical as voiced by our participants and others (Gregus et al., 2020).

Survey responses indicated that race and ethnicity were rated as the areas of highest satisfaction and an area of highest need in the US. These findings are consistent with a survey of clinical psychology trainees a decade earlier, where students also reported highest satisfaction with training in race and ethnicity (Green et al., 2009). CC training in USA has traditionally focused on awareness of racial biases, knowledge pertaining to various racial/ethnic groups and skills in working with persons of color. Consequently, trainees rate their training in these areas as highly satisfactory. At the same time, systematic racism, discrimination, and racial disparities in health and education continue to pervade US society (Krieger, 2020), and there is increasing awareness of these issues, which may lead to identifying race and ethnicity as an area of need for more training.

*Recommendations* As the US becomes increasingly diverse, training programs are responding to the call to become diverse with more faculty and students who are persons of color, members of LGBQ+ community, and/or gender non-binary individuals. As a result, the traditional model of teaching White, straight cisgender male or female therapists to competently work with clients of color, or sexual and gender minority clients is no longer applicable for trainees with diverse intersecting identities. Training frameworks for CC need to incorporate the experiences of trainees with minoritized identities and how to best address them (e.g., how does a trainee respond to a racial, sexual, or gender-identity-based microaggression from a client or a supervisor?).

Further, graduate training programs should consider integrating CC throughout the curriculum including core clinical courses such as assessment, psychopathology, and intervention by weaving in readings and class discussions about bias, privilege, and marginalization, as well as varying idioms of distress and explanatory models, applicability of standardized tests for various subgroups, need for adaptations of empirically supported interventions, and interventions specifically developed for a particular demographic group. Barriers to such infusion (e.g., insufficient time, prioritizing other topics, lack of faculty competence) need to be addressed through innovative solutions. Coursework should avoid reinforcing stereotypes or overgeneralizations pertaining to various identity groups by using an intersectionality perspective that considers intersections of identities based on age, education, gender identity, language, religion, sexual orientation, social class, and other dimensions that contribute to varying levels of privilege and marginalization. In addition to race and ethnicity, sexual orientation, social class, and refugee status were identified as areas where further training was needed, and thus, these dimensions should be addressed in CC training. Finally, training programs should incorporate periodic evaluation of CC of their trainees in the areas of multicultural beliefs and attitudes, knowledge, skills, and advocacy to ensure that trainees are meeting competencies (Jones et al., 2013).

### Continuing Professional Development Regarding CC for Faculty and Practitioners

Faculty participants in both countries reported learning CC primarily through research and clinical experiences, suggesting that many faculty members may not have had exposure to theoretical foundations of CC through coursework in their own training programs. Graduate training programs should consider opportunities for continued professional development for faculty with respect to CC, for example, through faculty development programs that involve cultural immersion experiences, experiential learning, and self-reflective activities. Similarly, for practitioners, both those who did not receive formal training in CC as well as those who did, continuing education in CC needs to be considered mandatory as it is integral to providing ethical care to diverse clients.

### Limitations and Future Directions

Trainees in clinical or counseling psychology graduate programs with privileged social identities (cisgender women or men, straight, White in USA; cisgender, straight, Hindu in India) are overrepresented in our data, and thus, perspectives of trainees with minoritized social identities may not be as well represented. Given the finding that CC training in the US may be primarily geared toward White, straight, cisgender trainees, it is critical to explore the experiences of students, faculty, and practitioners with minoritized racial, ethnic, gender and sexual identities. Our results may also not generalize to other areas of health service psychology (e.g., school psychology). Although our overall survey sample sizes in both countries were adequate, faculty and practitioner samples were somewhat small. Future research with larger samples of faculty, students, and practitioners that assesses training in CC, competencies gained, and the extent to which individuals incorporate CC training into their clinical practice is needed. In addition, longitudinal research that follows graduate students from their first to final year of the graduate program and beyond may be beneficial in assessing change in CC as a result of training.

Despite limitations, the current findings contribute to scarce literature on CC in health service psychology graduate programs in diverse societies around the world. The findings highlight the relevance of infusion of CC through theoretical frameworks, clinical practice experiences with diverse clients, as well as life experiences in facilitating the development of CC, while underscoring that trainees need to continue to devote time and efforts toward life-long learning. Results may inform training programs in both countries to ensure that CC training is responsive to trainees’ needs and prepares them to engage in culturally competent care.
